# The importance of genotype identity, genetic heterogeneity, and bioinformatic handling for properly assessing genomic variation in transgenic plants

**DOI:** 10.1186/s12896-018-0447-9

**Published:** 2018-06-01

**Authors:** Jean-Michel Michno, Robert M. Stupar

**Affiliations:** 1 Bioinformatics and Computational Biology Program, University of Minnesota, Minneapolis, MN USA; 2 Department of Agronomy and Plant Genetics, University of Minnesota, 1991 Upper Buford Circle, 411 Borlaug Hall, Saint Paul, MN 55108 USA

**Keywords:** Soybean, Transgenic, Bioinformatics, Heterogeneity

## Abstract

**Background:**

The advent of –omics technologies has enabled the resolution of fine molecular differences among individuals within a species. DNA sequence variations, such as single nucleotide polymorphisms or small deletions, can be tabulated for many kinds of genotype comparisons. However, experimental designs and analytical approaches are replete with ways to overestimate the level of variation present within a given sample. Analytical pipelines that do not apply proper thresholds nor assess reproducibility among samples are susceptible to calling false-positive variants. Furthermore, issues with sample genotype identity or failing to account for heterogeneity in reference genotypes may lead to misinterpretations of standing variants as polymorphisms derived de novo.

**Results:**

A recent publication that featured the analysis of RNA-sequencing data in three transgenic soybean event series appeared to overestimate the number of sequence variants identified in plants that were exposed to a tissue culture based transformation process. We reanalyzed these data with a stringent set of criteria and demonstrate three different factors that lead to variant overestimation, including issues related to the genetic identity of the background genotype, unaccounted genetic heterogeneity in the reference genome, and insufficient bioinformatics filtering.

**Conclusions:**

This study serves as a cautionary tale to users of genomic and transcriptomic data that wish to assess the molecular variation attributable to tissue culture and transformation processes. Moreover, accounting for the factors that lead to sequence variant overestimation is equally applicable to samples derived from other germplasm sources, including chemical or irradiation mutagenesis and genome engineering (e.g., CRISPR) processes.

**Electronic supplementary material:**

The online version of this article (10.1186/s12896-018-0447-9) contains supplementary material, which is available to authorized users.

## Background

The process of genetic transformation typically involves inserting DNA sequences originating from one species into the genome of another species. This tool has been used to add traits into crop species, such as herbicide tolerance in soybean and root worm tolerance in corn [1–4]. The commercialization of transgenic products is subject to tight regulation, as transgenic strains must undergo intense safety testing before being brought to market [5]. The testing phase involves confirmation of the intended trait encoded by the transgene, and confirmation that the transgenic plant does not have unintended consequences that may be detrimental to the environment or to the consumer [6]. Adverse effects are generally characterized in two categories: effects from the transgene itself, and effects that arise from mutations resulting from gene insertion or the tissue culture process. As a result, safety testing ensures that unintended DNA-level changes are not present in commercialized products [7, 8].

With the recent revolution in high-throughput sequencing technology, there is now increased interest in understanding the molecular nature of transgenic events, and identifying possible safety implications of unintended molecular changes that may result. This information may be useful in assessing the likelihood that a particular event will express the intended trait(s) without detrimental unintended effects.

Molecular studies have previously characterized the effects of transgenesis in several different plant species, focusing on the sequence changes at transgene integration sites [9, 10] and/or the sequence changes genome-wide [11–19]. While no clear consensus has emerged, studies utilizing sequence-level resolution have reported a range of possible sequence changes in transgenic plants, including frequent observations (e.g., small deletions occuring adjacent to the integration site) and less frequent occurances (e.g., translocations between chromosomes).

A curious discrepancy in genome-wide sequence polymorphisms has been observed in recent resequencing studies of transgenic soybean. One study, published by our group [20], resequenced two independent transgenic T1 plants, and respectivley found only two and 18 single nucleotide polymorphisms (SNPs) genome-wide (along with deletions adjacent to the integrated transgene, as has been previously observed in other plant transformation studies). In contrast, Lambirth et al. [21, 22] reported high rates of molecular variation among transgenic soybean plants, both in terms of transcriptomic changes and DNA sequence changes. The authors analyzed RNA-sequencing (RNA-seq) data on families from three different transgenic events and reported thousands of sequence variants per plant, focusing on SNPs and small insertion-deletion (indel) variants. They reported tens of thousands of sequence variants in these plants, including approximately 1000 to 7700 variants that were unique to each of the three event series. This contrast between studies is even more surprising considering that Anderson et al. [20] searched genome-wide while Lambirth et al. [22] searched only the transcribed portion of the genome. Both groups were studying the same species (soybean) transformed by similar methods (*Agrobacterium*-mediated transformation of cotyledonary nodes) [23] and resequenced using similar chemistries (Illumina short-read).

Given the importance and real-world relevance of this topic, it is imperative to resolve the discrepancy between the Anderson et al. [20] and Lambirth et al. [21, 22] studies. We are not aware of any transgenic resequencing studies that have reported mutations rates similar to those published by Lambirth et al. [22]. Therefore, the current study focuses on a reanalysis of the Lambirth et al. [22] dataset, applying a more stringent analytical pipeline. The outcome of this reanalysis demonstrates that the Lambirth et al. [21, 22] studies overestimated the transcriptional and DNA sequence variation in the transgenic plants. These findings provide insight into the importance of identity preservation of genotypes, awareness of genomic heterogeneity within cultivars, and leveraging bioinformatics filters and replicated data as a way to minimize false positives.

## Results and discussion

### Primary source of variation in transgenic event series 764: Incorrectly identified genetic background

Lambirth et al. [21, 22] performed RNA-seq analyses of 27 transgenic plants, including nine individuals each selected from three different transgenic series known as ST77, ST111, and 764. They reported that all three of these transgenic series were developed in the genetic background of cultivar ‘Williams 82’. As a control, they also performed RNA-seq on nine individuals of ‘Williams 82’, thus resulting in a total of 36 RNA-seq samples in the study. As ‘Williams 82’ was also the genotype used to develop the soybean reference genome [24], all of the mutations reported by [22] were identified simply by comparing their transcriptome sequence to the reference genome. The authors reported surprisingly high mutation frequencies in both the transgenic and control plants, particularly the 764 transgenic event series. As de novo mutations caused by the tissue culture or transgenesis pathway are expected to be unique to a given event, the authors calculated the number of unique event-specific mutations in each series compared to the other groups/series in the study (i.e., the number of mutations in one series that is not shared by the other two series of transformants or the control ‘Williams 82’ plants). They reported a unique polymorphic SNP count of 981 in event ST77, 927 in event ST111, and 7717 in event 764. This discrepancy matched their earlier analysis of gene expression variation among three series, where series 764 exhibited much greater expression variation as compared to controls than did the other two transgenic groups [21].

Two findings in the Lambirth et al. [22] mutation analysis stand out: (1) The SNP frequencies were much higher than other similar studies of soybean [20] and model plant species [11–19], particularly considering that only the transcribed portion of the genome was being analyzed; (2) Even with the generally high mutation rates reported, the 764 series is still an outlier. To cross-validate the findings of this analysis, we downloaded and reanalyzed the raw RNA-seq data from these studies.

Using the GATK Best Practices workflow [25, 26], we re-generated polymorphic SNP lists from all 36 samples of RNA-seq data used [21, 22]. As stated above, de novo SNPs generated by tissue culture or transformation would be expected to be unique to each respective transgenic event. Therefore, we focused our analysis on SNPs that were unique to only one of the four groups (e.g., SNPs observed as an alternative base in one transgenic series, while matching the reference genome sequence in the other two transgenic series and the ‘Williams 82’ controls). Given that the transgenic plants were self-pollinated for several generations after transformation, the SNPs derived from the tissue culture or transformation process are expected to be predominantly homozygous. Therefore, we filtered our initial lists for homozygous SNPs that are uniquely polymorphic relative to the reference genome, compared to the other transgenic lines and ‘Williams 82’ controls (Additional file 1: Figure S1). This analysis and filtering pipeline differed from the Lambirth et al. [22] pipeline in at least four critical ways: (1) The GATK Best Practices workflow imposed a higher standard for calling variants (see Methods section); (2) we did not include heterozygous calls; (3) we did not include heterogeneous SNPs among the nine samples of any group (the three transgenic series or controls); (4) we required at least six out of the nine samples within each group to exhibit the same homozygous base call.

The analysis and filtering pipeline described above was designed to prevent false-positive SNP calls. Nevertheless, the pipeline was able to detect nearly 10,000 SNPs among the transgenic samples (Table 1, Additional file 2: Table S1). However, the distribution of SNPs among the genotypes was substantially different than what was reported previously [22]. Almost all of the unique SNPs that we identified were found in transgenic series 764 (9738 out of the 9884 SNPs). Meanwhile, only 143 and 3 SNPs, respectively, were identified in ST77 and ST111 (Table 1).

**Table 1:** Number of SNPs identified as unique for each transgenic line based on reanalysis of the RNA-Seq dataset

	764	ST77	ST111	Williams 82
“Unique” SNPs found in the whole RNA-Seq dataset	9738	143	3	0
“Unique” SNPs found in the RNA-Seq dataset that overlap with 50 k SNP positions	525	11	0	0

**Fig. 1:**
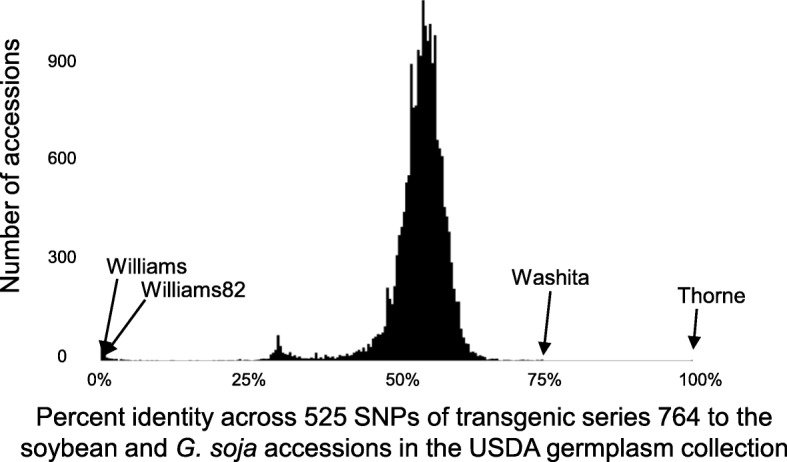
Reanalysis of series 764 reveals that its genetic background comes from genotype ‘Thorne’ rather than genotype ‘Williams 82’. 525 SNPs were identified that met two criteria: (1) they were consistently polymorphic between series 764 plants and the ‘Williams 82’ reference genome in the RNA-seq dataset; (2) they were previously genotyped across the USDA germplasm [27]. A comparison of these SNPs to the all of the accessions in the USDA soybean accessions revealed ‘Thorne’ as a near-perfect match (99.2% identity), with a substantial gap to the next closest match (Washita at 74.2%). The reanalysis also confirmed that this panel of SNPs is completely polymorphic between the 764 series and ‘Williams 82’ (0% match)

We postulated that the discrepancy exhibited by the 764 series might have resulted from experimental error rather than biological factors. To test this, we compared the list of SNPs we generated (Table 1, Additional file 2: Table S1) with a list of pre-ascertained SNPs that were previously used to genotype the entire USDA soybean germplasm collection [27]. We found that 525 of the SNPs that were unique to series 764 also matched the genome positions on the pre-ascertained SNP list (Table 1, Additional file 2: Table S1). We compared the SNP profile of these 525 SNPs for series 764 with all of the accessions in the USDA collection. One genotype, cultivar ‘Thorne’ (PI 564718) [28], was a nearly perfect match to series 764 (521 of the 525 SNPs match; Fig. 1). The four SNPs that did not match between series 764 and ‘Thorne’ were clustered together between positions ~ 4.9 Mb and ~ 5.9 Mb on chromosome 15. It is likely that this interval on chromosome 15 represents a region of genetic heterogeneity between the individual of ‘Thorne’ used for transformation in the development of the 764 event and the individual(s) of ‘Thorne’ sampled for the USDA genotyping effort [27]. While the series 764 profile was a 99.2% match to ‘Thorne’ across the 525 SNPs, the next closest match was ‘Washita’ (PI 618809) [29], which was only a 74.2% match. Both ‘Williams’ and ‘Williams 82’ had a 0% match rate to the 525 SNPs in the 764 series (Fig. 1), as would be expected because the reference genome is based on ‘Williams 82’ and these SNPs were initially identified as polymorphic between the 764 series and the reference genome.

The clear conclusion from this analysis is that series 764 was developed in ‘Thorne,’ rather than ‘Williams 82’. ‘Thorne’ is commonly used for soybean transformation (e.g., [23]). It is clear that the high polymorphism rate reported in event series 764 is not an unintended consequence of tissue culture or transgenesis. Instead, the majority (if not all) of the variation reported in this line is simply standing variation that exists between ‘Thorne’ and ‘Williams 82’. This statement can be applied to all previous reports of variation observed between these plants, including gene transcription [21], mutations [22], or any other characteristic.

### Source of variation in transgenic event series ST77: Genetic heterogeneity between different individuals of ‘William 82’

The relatively lower polymorphism rates found in the reanalysis of S77 and S111 compared to that of 764 (Table 1) indicated that these groups are likely derived from the ‘Williams 82’ background. However, standing variation can persist within soybean cultivars [30], as the breeding process typically involves bulk harvesting of breeding populations prior to full fixation of homozygosity through single seed descent. Therefore, most soybean cultivars are expected to exhibit slight differences from plant to plant [31, 32], as heterogeneous sub-lines fix different haplotypes within relatively small (but sometimes large) genomic intervals. For example, previous genotyping of four different ‘Williams 82’ sub-lines revealed specific regions of genomic variation on chromosomes 3, 7, 15 and 20 [30].

**Fig. 2:**
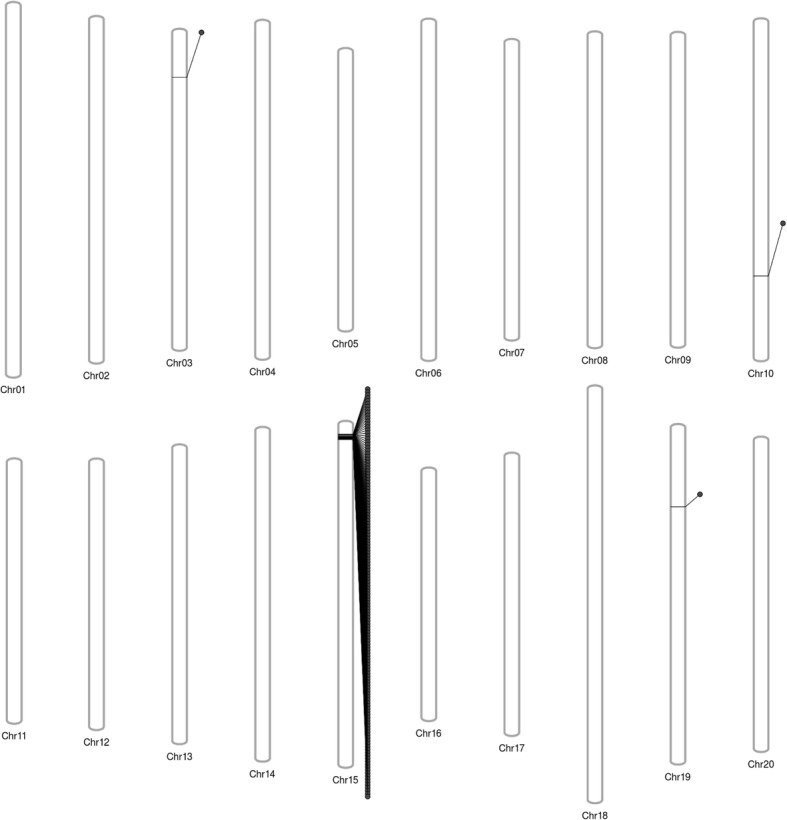
Distribution of unique SNPs across transgenic series ST77. The distribution of the 143 unique SNPs identified in ST77 is shown among the 20 chromosomes. Almost all of the ST77 SNPs (140 out of 143) cluster at a single locus on chromosome 15, which is a typical signature of genetic heterogeneity among the ‘Williams 82’ parental lines used in this study. The clustering of SNPs at specific, rather than random, positions is indicative of heterogenous standing variation that has previously documented in the ‘Williams 82’ cultivar [30]

It is relatively intuitive to identify genomic heterogeneity between sub-lines of a cultivar, as sub-lines will show nearly complete homogeny throughout the genome, interrupted by specific regions with (sometimes dense) clusters of polymorphisms. We investigated whether the 143 SNPs identified in our reanalysis of group ST77 could be explained by this type of standing heterogeneity between the ‘Williams 82’ controls used in the study and the ‘Williams 82’ individual that was used for the original ST77 transformation event [21, 22]. Indeed, 140 of the 143 SNPs and all 16 indels were clustered at a single locus between positions 1.4 Mb and 2.2 Mb on chromosome 15 (Fig. 2). This cluster overlaps with a previously reported region of heterogeneity in ‘Williams 82’ [30]. These results suggest that these variants are not associated with transgenesis, but represent natural standing heterogeneity between the ‘Williams 82’ plant used to generate the ST77 transformation event and the ‘Williams 82’ individuals used as controls by Lambirth et al. [22].

Therefore, after filtering for genotype identity and background heterogeneity, we found three SNPs each in S77 and S111 that could not be explained by these factors. Follow-up analysis of S77 revealed one SNP within an intron, one synonymous SNP within an exon, and one non-synonymous SNP within an exon (M to V amino acid change in the sixth exon of Glyma.10G150500). Analysis of S111 revealed two SNPs within introns, and one non-synonymous SNP within an exon (T to G amino acid change in the fourth exon of Glyma.04G134800).

### Source of variation in all transgenic series: Bioinformatics handling and threshold parameters

The previous two sections addressed our reanalysis of RNA-seq data [21, 22], focusing on the subset of unique SNPs and indels within any one transgenic series. However, the majority of the analysis reported, discussed and interpreted in the Lambirth et al. [22] paper (including the base substitution profile, the predicted effect of each polymorphism, and gene ontology enrichment analysis) used the original full set of SNPs and indels identified, rather than the “unique” subset. Hence it is necessary to focus on the factors that inflated the overall higher number of SNPs and indels discovered by their bioinformatic pipeline. While we would expect the authors to identify polymorphisms due to the reasons outlined in the previous sections (e.g., the ‘Thorne’ background of series 764 and the genetic heterogeneity between ST77 and the control ‘Williams 82’ plants), the reported polymorphism counts were unexpectedly high. For example, the plants in the 764 series averaged 38,188 SNPs and 2390 indels per plant. This number will be higher than the other two transgenic series because it is the ‘Thorne’ genetic background. However, the ST77 series averaged 21,666 SNPs and 1829 indels, and the ST111 series averaged 20,208 SNPs and 1750 indels. Furthermore, the untransformed ‘William 82’ control plants exhibited counts of 20,707 SNPs and 1863 indels. Therefore, this section is devoted to addressing the sources of these high estimates.

We retrieved the variant calls for each of the 36 samples used in their analysis (http://de.iplantcollaborative.org/dl/d/533570A3-1EFB-4864-B9A9-9D82F17E09A8/snpeffgenes.zip). Initial analyses of genotype calls revealed that there was a higher number of heterozygous variants than homozygous variants for the alternate allele compared to the reference genome. ST77 and ST111 were respectively advanced to the T8 and T4 generation before sequencing. We can estimate the expected proportion of heterozygous variants in these generations if we assume the following: all of the mutations induced by transgenesis were heterozygous in the T0 generation, the variants are not subject to segregation distortion, and the variants have negligible effects on organismal fitness. Under these assumptions, we would expect approximately 0.39% of the ST77 variants to be heterozygous at the T8 generation, and 6.25% of the ST111 variants to be heterozygous at the T4 generation. However, the retrieved data showed that 50.21 and 48.62% of the variants were called as heterozygous for ST77 and ST111, respectively. The proportion of heterozygous variants were far higher than what was expected, and were most likely false positives resulting from the analysis method.

**Fig. 3:**
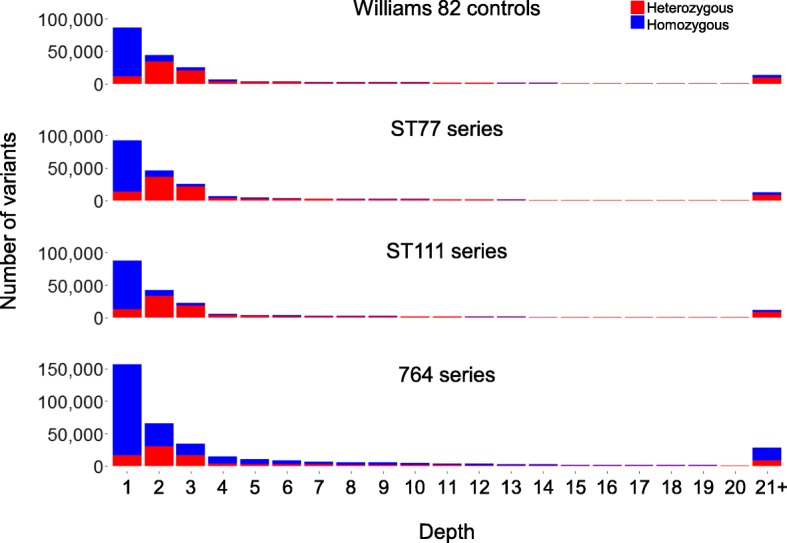
Depth of sequence coverage for all polymorphic variants (SNPs and indels) called in the Lambirth et al. [22] study. The polymorphic calls shown here were made between each sample and the reference genome ‘Williams 82’, without consideration for the uniqueness of the call among series or reproducibility among different plants within the series. Homozygous calls are shown in blue and heterozygous calls are shown in red. Each bar sums the number of polymorphisms across the nine plants that were called at each read depth (e.g., we are showing the ~ 211,448 total variants called in series ST77 across the nine plants; ST77 averaged 23,494 variants per plant). Note the relatively larger peak in the 21+ category for the 764 series compared to the other series; many of these (mostly homozygous) calls likely represent standing variants between lines ‘Thorne’ and ‘Williams 82’. The 21+ peaks in the other three groups (ST77, ST111, and ‘Williams 82’ controls) may derive from various factors, most obviously the clusters of variants that are found within heterogeneous regions of different sub-lines of ‘Williams 82’

We further investigated whether the authors filtered their variants for read depth and/or quality. Although read depth alone is not sufficient to determine whether a variant is real, calls based on low read depth are more likely to be false positives than calls based on higher read depths. False positives can arise from reads that map poorly to the genome, or bases that are of low quality at the site of a polymorphism. When analyzing the depth of variant calls for all 36 samples in the study, 43.2% of variants were called at a depth of one read, and 20.2% of variants were called with a depth of two reads (Fig. 3). Similarly, when analyzing the distribution of quality scores across all 36 samples, 55.3% of variant calls had a quality score of 10 or lower (Additional file 1: Figure S2). A quality score is represented on a log-based Phred scale where, for example, a quality score of 10 indicates that there is a 10% chance of the variant being incorrect and a quality score of 20 indicates that there is a 1% chance of the variant being incorrect. Further investigation into the authors’ methods revealed that the variant calls lacked any type of depth or quality filter. This further reinforces the likelihood that a large portion of these variants at low depth and quality are most likely false positives.

**Fig. 4:**
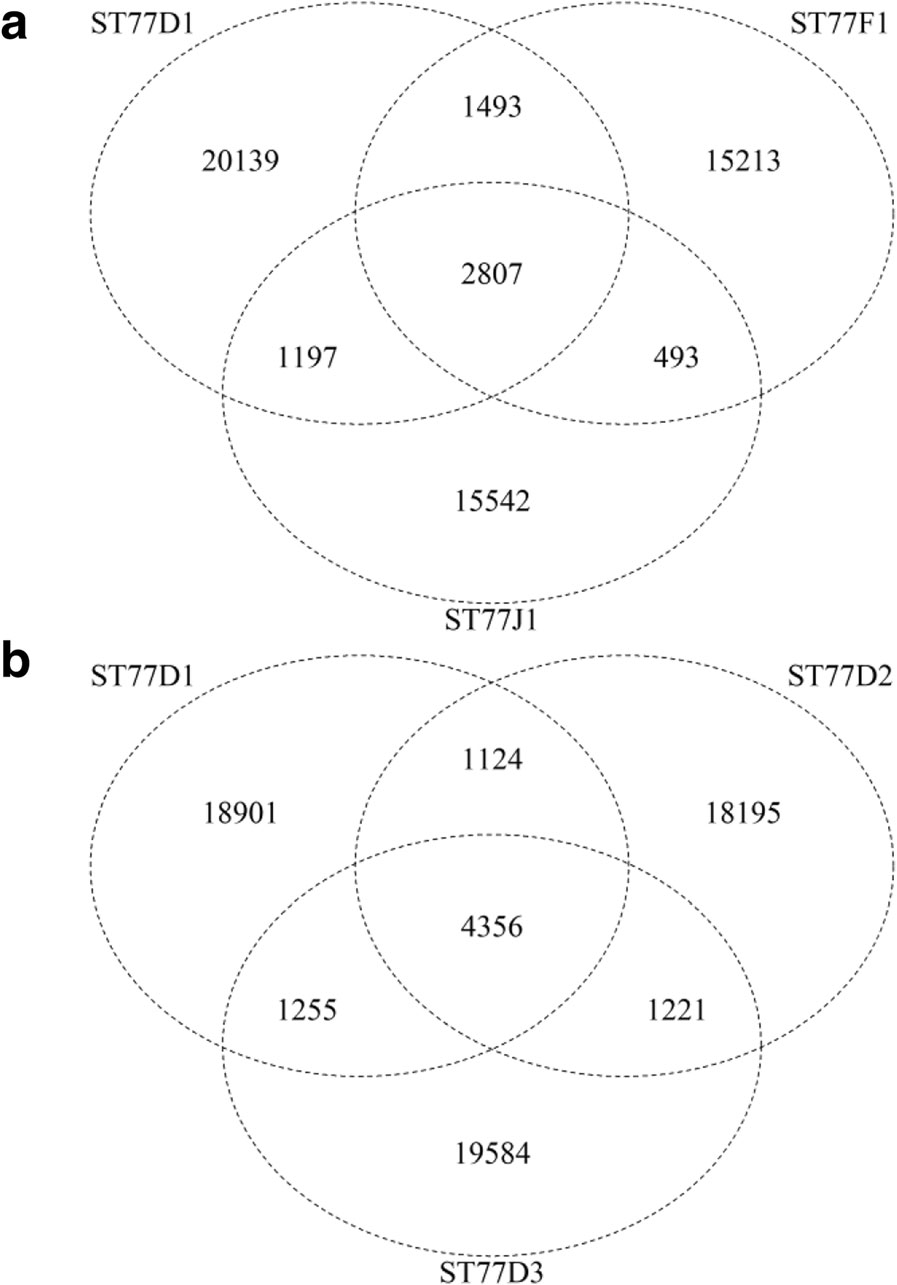
Number of overlapping polymorphisms in the Lambirth et al. [22] study. **a** Venn diagram showing of the number of sequence variants alternate to the reference genome that overlapped between three T8 individuals derived from transgenic event ST77. Heterozygous and homozygous alternate calls are not differentiated in this analysis. **b** A similar Venn diagram of the number of polymorphism that overlapped between the T7:8 siblings in the ST77D family

The experiments in these studies [21, 22] included the sequencing of nine samples per transgenic series (or the ‘Williams 82’ controls), consisting of three sibling seeds taken from three plants each. As mutations induced by transformation or tissue culture would presumably occur in the T0 generation, one would expect the vast majority of these loci to be fixed as homozygotes by the T4-T8 generations. Therefore, it may be intuitive to exclude any variants that were not observed in all three siblings. While the authors reported on average ~ 20,000 SNPs and ~ 1800 indels per individual plant for ST77, ST111, WT, and ~ 40,000 SNP’s and ~ 2400 indels per individual plant for 764 compared to the reference genome, the majority of variants were detected as polymorphic in only one of the 36 samples in the study. Figure 4a shows a comparison of the variants from three selected ST77 plants, each derived from a different T_7_ individual. In this case, over 20,000 variants were called for each plant, but only 2807 of the variants were common across all three plants (Fig. 4a). Similar findings were observed for the ST77 “D” series siblings (all derived from a T_7_ plant designated as “D”), in which a relatively small proportion (4356 out of 64,636) of the variants were in common to all three siblings (Fig. 4b). These trends were observed across all sibling groups in the study (Additional file 1: Figure S3). Series 764 exhibited a greater proportion of variants shared among the siblings, which would be expected for a plant from a different genetic background than ‘Williams 82,’ i.e., these plants have more “true” sequence variants that can be faithfully detected among the different siblings.

Another indication of the high frequency of false positives called in the Lambirth et al. [22] study relates to the structure of the indels that were called as polymorphic. Of the 70,486 indels that were called, 52.9% of them were heterozygous and 59.6% of them had a read depth of 3 or less. Interestingly, all of the indels reported in the study exhibited polymorphisms that were either 1 bp insertions (22,809 calls), 2 bp insertions (8480 calls), 1 bp deletions (13,427 calls) or 2 bp deletions (25,770 calls). The high number of only 1- or 2-bp indels (Additional file 2: Table S2) are likely a consequence of the read mapping software and bioinformatics pipeline used [33].

## Conclusions

In the present study, we re-examined an existing data set that was previously used to report high mutation counts from three transgenic plant series. We identified three major factors that inflated the estimates of molecular variation in the transgenic plants from these studies. These factors included residual heterogeneity, genotype misidentification, and insufficient data filtering. The issue of genotype identity is obvious and intuitive, but requires caution, both for those handling and maintaining the materials (e.g., seeds, tissue, DNA) and those handling the computational analysis. Errors in genotype identity can be diagnosed using strictly molecular approaches, but situations where the identity of the material has been compromised or misinterpreted can be problematic (see commentaries [34, 35]). The issue of genetic heterogeneity within lines and seed stocks can create more subtle complications in analysis, as has been documented in the soybean line ‘Williams 82’ [30]. When properly accounted for, heterogenetity does not disrupt accurate analysis and interpretation. However, when not properly accounted for, this issue may be problematic in assessing genomic, transcriptomic, and other types of variation. Within-line genetic heterogeneity can be an issue in many species, particularly those in which a reference genome is presumed to be perfectly representative of every individual in the seed stock. Lastly, data handling can be a major source of variation leading to inflated variant calls. Informatics pipelines generate large data sets, and users should be aware of quality control measures, and commonly used filtering parameters. Furthermore, experimental designs that provide replicated samples or comparisons among near-isogenic materials (e.g., the sibling lines discussed in this study) can be used to further differentiate the high-confidence and low-confidence variant calls.

While the present reanalysis focused specifically on comparisons between transgenic lines, all the factors addressed in this paper need also be considered when conducting any type of expression and/or genomic comparisons. This includes studies that focus on the effects of mutagenesis, on-target and off-target effects of genome engineering technologies, assessments of standing/natural variation, or other comparisons of germplasm sources. This is particularly true for experiments on materials within the realm of biotechnology, as the findings may be used to inform regulatory agencies about the intended and unintended consequences of using these technologies. Evaluation for the presence of unintended changes at the DNA level continues to be a part of the safety evaluation for transgenic plants, and whole-genome sequencing has been proposed as a tool for this purpose [36]. However, technical issues may make this problematic in crop species, which have complex, highly variable, and often heavily duplicated genomes. Furthermore, as demonstrated by the present study, the analysis and interpretation of whole-genome sequencing data may be inconsistent among research groups. While Lambirth et al. [22] reported high rates of mutation in transgenic soybean lines, our reanalysis of their data concluded that there are relatively few sequence variants detected in these lines that might be attributed to the transformation process. It will be difficult to standardize a regulatory methodology that accounts for every complication that will arise across research groups and species (e.g., standing genetic heterogeneity within a parental seed stock) that may be incorrectly attributed to the genetic transformation process.

## Methods

### Variant and indel detection

RNA-seq from [21] was downloaded from the National Center for Biotechnology Information Sequence Read Archive using project number PRJNA271477 and reanalyzed as described below. Sequencing adapters and low-quality bases were removed using Cutadapt with minimum read length set to 40 and quality cutoff set to 20 [37]. Using the GATK Best Practices workflow for RNA-seq [25, 26], reads were aligned to assembly version two of the reference genome (Wm82.a2) from www.soybase.org using the STAR aligner [38]. Read-group identifications were added and duplicate reads were marked using Picard tools. Reads were then split into exon segments, overhanging intronic segments were hard clipped, and mapping qualities were reassigned using the SplitNCigarRead tool from the GATK Genome Analysis Toolkit with -RMQF set to 255 -RMQT set to 60 and enabling the -U ALLOW_N_CIGAR_READS flag [39]. SNPs and indels were called using GATK HaplotypeCaller with the -dontUseSoftClippedBases flag and -stand_call_conf set to 20. The resulting VCF file was then split into separate files for SNPs (Additional file 3) and indels (Additional file 4) and then filtered using VariantFiltrations from the Genome Analysis Toolkit with parameters set to window of 35, cluster of 3, filter parameters of FS > 30, and QD < 2.0 for SNPs. Similar parameters were used for indel filtration, except FS filter was set to > 200 for all 36 samples. Variants that passed filtration were then used for downstream analysis.

### Accession identification

Genotype calls from the filtered SNP list were extracted using a custom python script then loaded into R statistical software. The dataset was filtered for homozygous SNPs that are uniquely polymorphic to the reference compared to the other transgenic lines and ‘Williams 82’ controls. SNPs were removed from the analysis if there was more than 33% missing data for a given line and if there was no consensus genotype call between plants and replicates (Additional file 1: Figure S1). The resulting SNPs were used to identify positions that overlapped within the SoySNP50k iSelect BeadChip [27] VCF file using the Wm82.a2 coordinates downloaded from www.Soybase.org. SNP calls for each of the 20,087 accessions in the 50 k dataset were compared to the SNP calls for the 764 series to identify the accession with the highest level of SNP identity.

### Analysis of data from previous studies

The Lambirth et al. [22] supplementary data was downloaded from http://de.iplantcollaborative.org/dl/d/533570A3-1EFB-4864-B9A9-9D82F17E09A8/snpeffgenes.zip, and each of the 36 samples VCF files were parsed for depth, quality, and genotype information using a custom python script.

### Software and figures

Parallelization of commands was run using GNU parallel. Data that was generated using R statistical software was plotted using the ggplot2 package [40]. The genome distribution of SNPs was created by using Phenogram [41].

### Data availability

Software versions, options, thresholds, workflow details and custom scripts can be found at https://github.com/MeeshCompBio/The_Other_WPT_Study.

## Additional files

Additional file 1: **Figure S1.** Pipeline to identify the background genotype of 764. **Figure S2.** Quality scores for all polymorphic variants (SNPs and indels) called in the Lambirth et al. [22] study. **Figure S3.** Number of overlapping polymorphisms in the Lambirth et al. [22] study within each of the 12 sibling families studied. (PPTX 1455 kb)

Additional file 2: **Table S1.** SNP calls resulting from the data filtering pipeline shown in Additional file 1: Figure S1, excluding the accession identification steps. The SNPs correspond to the top row in Table 1. **Table S2.** Indel calls resulting from the data filtering pipeline shown in Additional file 1: Figure S1, excluding the accession identification steps. (XLSX 2307 kb)

Additional file 3: Resulting raw variant SNP calls from GATK HaplotypeCaller. (VCF 49825 kb)

Additional file 4: Resulting raw variant indel calls from GATK HaplotypeCaller. (VCF 29397 kb)

## Abbreviations

CRISPR: Clustered Regularly Interspaced Short Palindromic Repeats
Indel: insertion-deletion
RNA-seq: RNA-sequencing
SNP: Single nucleotide polymorphisms
