# Supplementary material for: The importance of genotype identity, genetic heterogeneity, and bioinformatic handling for properly assessing genomic variation in transgenic plants
Source: BMC Biotechnol. 2018 Jun 1;18:38. doi: 10.1186/s12896-018-0447-9 (PMC5984819; doi:10.1186/s12896-018-0447-9)
Supplement: Supplementary file 1 — Figure S1. Pipeline to identify the background genotype of 764. Figure S2. Quality scores for all polymorphic variants (SNPs and indels) called in the Lambirth et al. [22] study. Figure S3. Number of overlapping polymorphisms in the Lambirth et al. [22] study within each of the 12 sibling families studied. (PPTX 1455 kb) [file 12896_2018_447_MOESM1_ESM.pptx]

## Slide 1
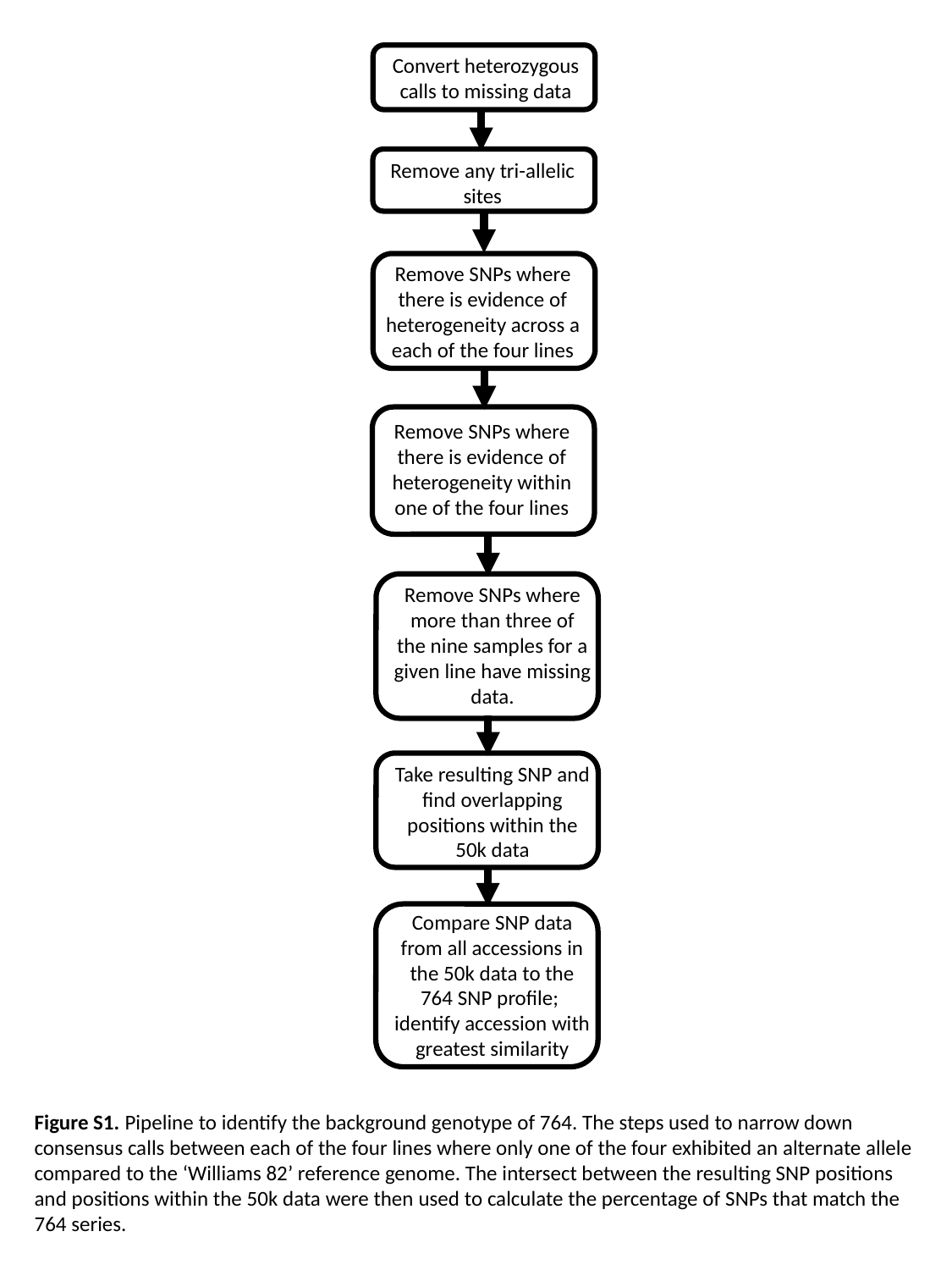

Convert heterozygous calls to missing data
Remove any tri-allelic sites
Remove SNPs where there is evidence of heterogeneity across a each of the four lines
Remove SNPs where there is evidence of heterogeneity within one of the four lines
Remove SNPs where more than three of the nine samples for a given line have missing data.
Take resulting SNP and find overlapping positions within the 50k data
Compare SNP data from all accessions in the 50k data to the 764 SNP profile; identify accession with greatest similarity
Figure S1. Pipeline to identify the background genotype of 764. The steps used to narrow down consensus calls between each of the four lines where only one of the four exhibited an alternate allele compared to the ‘Williams 82’ reference genome. The intersect between the resulting SNP positions and positions within the 50k data were then used to calculate the percentage of SNPs that match the 764 series.

## Slide 2
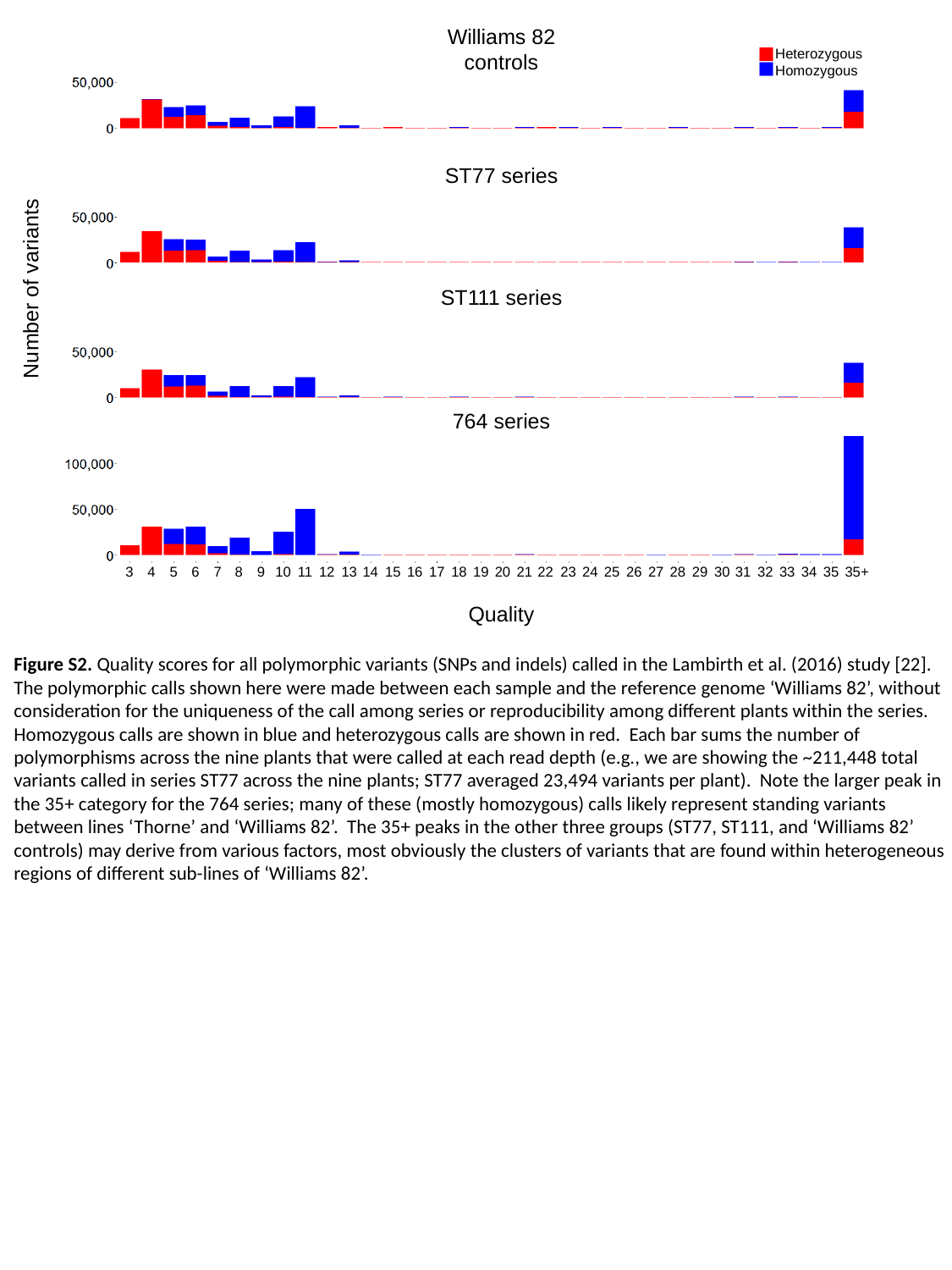

Williams 82 controls
Heterozygous
Homozygous
ST77 series
Number of variants
ST111 series
764 series
3
4
5
6
7
8
9
10
11
12
13
14
15
16
17
18
19
20
21
22
23
24
25
26
27
28
29
30
31
32
33
34
35
35+
Quality
Figure S2. Quality scores for all polymorphic variants (SNPs and indels) called in the Lambirth et al. (2016) study [22]. The polymorphic calls shown here were made between each sample and the reference genome ‘Williams 82’, without consideration for the uniqueness of the call among series or reproducibility among different plants within the series. Homozygous calls are shown in blue and heterozygous calls are shown in red. Each bar sums the number of polymorphisms across the nine plants that were called at each read depth (e.g., we are showing the ~211,448 total variants called in series ST77 across the nine plants; ST77 averaged 23,494 variants per plant). Note the larger peak in the 35+ category for the 764 series; many of these (mostly homozygous) calls likely represent standing variants between lines ‘Thorne’ and ‘Williams 82’. The 35+ peaks in the other three groups (ST77, ST111, and ‘Williams 82’ controls) may derive from various factors, most obviously the clusters of variants that are found within heterogeneous regions of different sub-lines of ‘Williams 82’.

## Slide 3
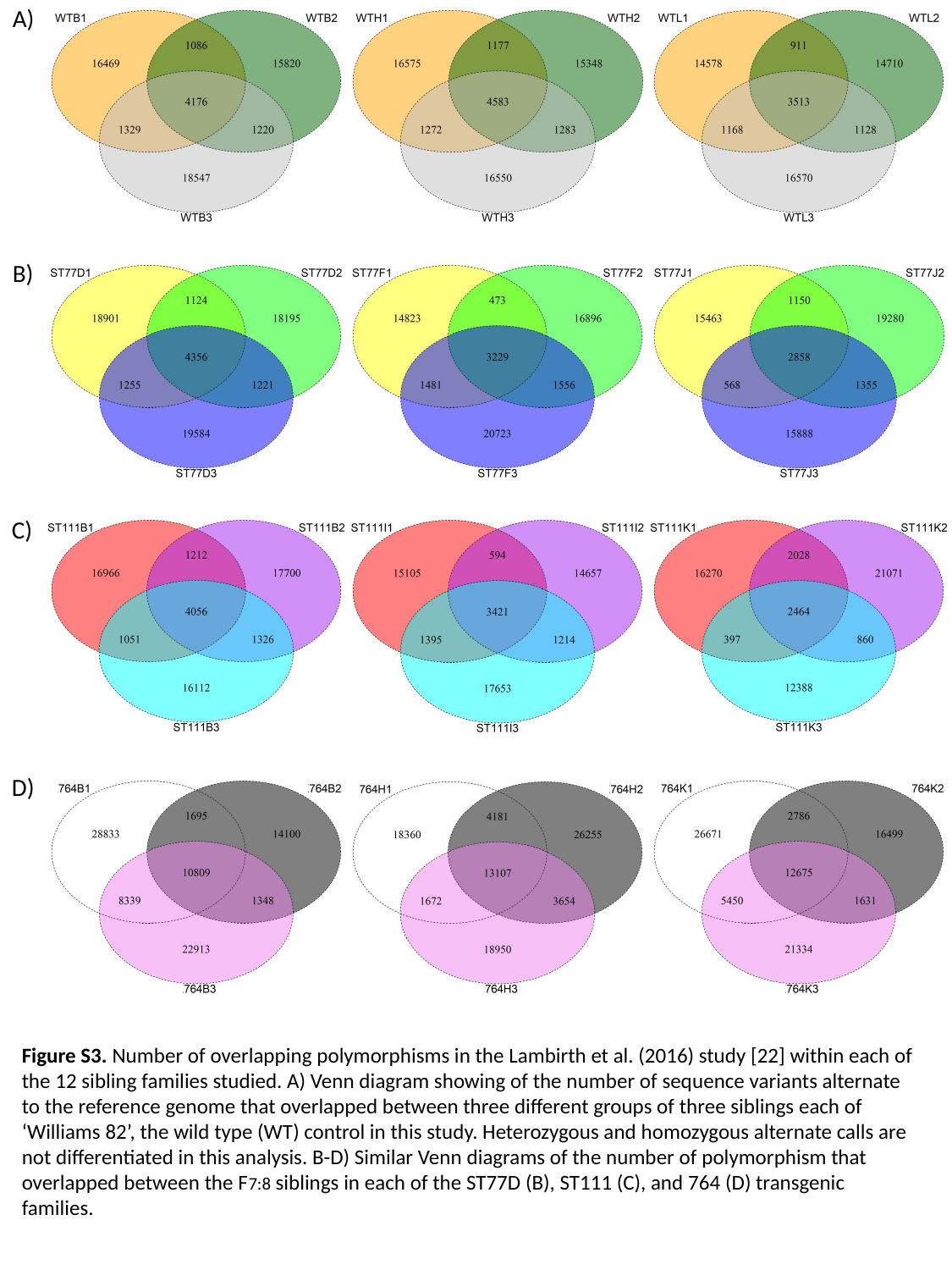

A)
B)
C)
D)
Figure S3. Number of overlapping polymorphisms in the Lambirth et al. (2016) study [22] within each of the 12 sibling families studied. A) Venn diagram showing of the number of sequence variants alternate to the reference genome that overlapped between three different groups of three siblings each of ‘Williams 82’, the wild type (WT) control in this study. Heterozygous and homozygous alternate calls are not differentiated in this analysis. B-D) Similar Venn diagrams of the number of polymorphism that overlapped between the F7:8 siblings in each of the ST77D (B), ST111 (C), and 764 (D) transgenic families.
